# One Health Perspective on the Enterotoxigenic *Escherichia coli* Diversity

**DOI:** 10.3390/microorganisms14061171

**Published:** 2026-05-22

**Authors:** Ricardo Rodríguez-Martínez, Jetsi Mancilla-Rojano, Sara A. Ochoa, Graciela Castro-Escarpulli, Ariadnna Cruz-Córdova, Juan Xicohtencatl-Cortes

**Affiliations:** 1 Departamento de Microbiología, Escuela Nacional de Ciencias Biológicas, Instituto Politécnico Nacional Carpio y Plan de Ayala, Col. Casco de Santo Tomás, Mexico City 11340, Mexico; www.birec@gmail.com (R.R.-M.); chelacastro@hotmail.com (G.C.-E.); 2 Laboratorio de Investigación en Bacteriología Intestinal, Hospital Infantil de México Federico Gómez, Mexico City 11340, Mexico; saraariadnah@hotmail.com; 3 Laboratorio de Investigación en Inmunoquímica, Hospital Infantil de México Federico Gómez, Mexico City 11340, Mexico; mancillajetsi@gmail.com

**Keywords:** enterotoxigenic *Escherichia coli*, colonization factors, reverse vaccinology, mRNA vaccines, environmental, one health, genomic surveillance, antigenic diversity

## Abstract

In this review, the virulence factors involved in enterotoxigenic *Escherichia coli* (ETEC) colonization and pathogenesis are analyzed, with an emphasis on colonization factors, enterotoxins and antigenic diversity as central challenges in vaccine development. ETEC remains a major cause of diarrhea worldwide, particularly in vulnerable populations. Despite extensive research, no broadly protective licensed vaccines are available largely because of antigenic heterogeneity and the limited understanding of immune correlates of protection. We identified critical knowledge gaps in antigen prioritization and host–pathogen interactions and translational limitations that have hindered vaccine success. We critically evaluated emerging platforms (including mRNA vaccines, nanoparticles, multiepitope strategies, and reverse vaccinology) for their potential to overcome variability and increase immunogenicity. We examined the roles of ecological environmental reservoirs associated with human and animal systems, in addition to antimicrobial pressure, in shaping ETEC evolution and vaccine effectiveness within a One Health framework; moreover, we propose an integrated approach that links genomic surveillance-based vaccine ecology and next-generation vaccine technologies to support adaptive immunogen design. This review provides actionable recommendations for the development of broadly protective and translationally viable ETEC vaccines from the One Health perspective.

## 1. Introduction

Enterotoxigenic *Escherichia coli* (ETEC) is responsible for approximately 220 million episodes of diarrhea worldwide each year, approximately 75 million of which occur in children younger than five years of age. Several studies have estimated that ETEC is responsible for 18,700 to 42,000 deaths each year among vulnerable groups [1,2,3,4]. This high mortality rate significantly affects at-risk populations worldwide. Although living standards have improved owing to better access to safe drinking water and sanitation, ETEC-related mortality rates remain significant, particularly among infants and children in low- and middle-income countries [1,2,3,4]. ETEC is among the earliest enteric pathogens encountered by infants in endemic countries, underscoring the urgent need for protective measures during the first 24 months of life. Furthermore, the treatment strategies used in many endemic settings have led to the emergence of antimicrobial resistance (AMR) among ETEC and other enteric pathogens. Importantly, prevention and treatment strategies are available, but their implementation in resource-limited settings remains severely limited [5,6,7]. In this context, the World Health Organization’s Product Development for Vaccines Advisory Committee (PDVAC) considers the development of effective vaccines against enteric pathogens, such as ETEC, a global public health priority [8,9].

Despite global efforts, ETEC remains a significant challenge, as no FDA-approved vaccines are currently available. Therefore, serious public health concerns exist, especially for children in low-income countries. The elucidation of new information related to the mechanisms of virulence and colonization in ETEC strains has significantly advanced the knowledge and development of viable solutions, such as vaccine. However, the development of potential vaccines against pathogens with highly variable virulence, such as ETEC, remains a significant challenge [10,11,12]. The lack of comprehensive basic and biomedical research and insufficient investment hinder scientists in their search for new vaccines. Currently, health policies are in their initial phase, and the generation of scientific knowledge, alongside practical interventions that address the urgent needs of disadvantaged populations, is being recognized and supported. Both whole-cell and subunit strategies targeting adhesins and enterotoxins have been investigated; however, identifying antigenic heterogeneity, geographic variability, and poor immunogenicity remains challenging. Recent advances include multiepitope structure-based platforms that increase multivalent and cross-protective immunity [13].

Despite several efforts, intestinal infections caused by pathogens that colonize the entire intestine, such as ETEC, remain a significant and urgent issue that has yet to be adequately addressed. ETEC thrives in diverse environments, including stagnant water and/or dams, where the conditions for bacterial growth and proliferation are optimal. These challenges are highlighted by the limited access to healthcare in affected areas and the need for coordinated efforts among public health authorities, global health organizations, research institutions, and funding agencies. The continued prioritization of evidence-based strategies to prevent ETEC infection as part of broader public health initiatives is essential [14,15]. In addition, ETEC strains play important roles in various populations and extend beyond acute episodes of intestinal disease. Recurrent infections caused by intestinal pathogens can have long-term consequences that significantly affect the cognitive and physical development of children, especially those in impoverished and vulnerable communities [3,16]. The health burden of ETEC infections is not merely a statistical concern; in Mexico, for instance, such diseases are often unreported, preventing the affected individuals from receiving timely and effective treatment [17]. This emerging health situation underscores the urgent need to improve the management and treatment of ETEC infections.

In this context, the One Health framework is not a model for cross-species vaccine development but rather an integrated perspective that acknowledges the interconnected roles of environmental reservoirs, antimicrobial pressure, genomic diversity, and socioecological determinants in influencing ETEC transmission and disease burden. Importantly, although many ETEC colonization factors (CFAs) demonstrate host-associated patterns, genomics and ecological surveillance under a One Health approach can help identify shared features and common targets, thereby facilitating vaccine development. Addressing the impact of ETEC on public health requires comprehensive strategies that extend beyond individual clinical practices and traditional disease management frameworks. Several epidemiological studies have highlighted the need to examine ETEC infections from a broader perspective that considers environmental exposure, antimicrobial resistance, and socioeconomic determinants. Within this One Health context, although host-specific CFAs are common between human and animal ETEC strains, certain conserved antigens and virulence mechanisms may offer opportunities for the development of cross-protective vaccine strategies. However, One Health is not only an integrated approach that acknowledges the interconnected roles of human health and dynamics but also a perspective that allows for a more accurate understanding of how factors such as water quality, sanitation, food production systems, and human behavior contribute to the persistence and spread of ETEC [18,19].

In this review, we focus on two main areas of study. First, we examine the challenges posed by the high variability of CFAs expressed by ETEC strains, which represent a significant constraint in the development of effective vaccines. Second, we highlight the relevance of the One Health approach in addressing the challenges posed by highly diverse pathogens (such as ETEC). In this framework, the use of emerging technologies, such as multiepitope platforms, reverse vaccinology, and mRNA vaccines, may offer significant potential to improve children’s health through the development and administration of effective vaccines against ETEC. This approach may prove promising and viable for breaking the cycle of damage caused by ETEC, as no vaccines are currently available against this pathogen. Several potential vaccines for ETEC are being developed and have shown encouraging progress [20,21].

ETEC CFAs are important virulence determinants; however, their high host specificity limits their effectiveness as the sole antigens for creating a universal cross-protective vaccine. Although the CFAs of humans and animals do exhibit some molecular similarities, differences in host-specific exotoxins and adhesins typically indicate that human strains do not cause disease in domestic animals (and vice versa). Nevertheless, the One Health approach is essential for understanding this issue, as environmental reservoirs, agricultural practices, and livestock systems play significant roles in ETEC transmission dynamics, which can help inform surveillance efforts.

This narrative review was conducted using a structured approach in accordance with PRISMA guidelines to ensure transparency in the identification and selection of studies. A comprehensive literature search was conducted across PubMed, Scopus, and Web of Science, covering studies published through 2025. Additionally, one seminal study from 1993 was included because of its high relevance. The search strategy employed both controlled vocabulary and free-text terms, including “Enterotoxigenic *Escherichia coli*” (ETEC), “colonization factors” (CFA), “virulence,” “vaccines,” “genomics,” and “One Health.” Boolean operators (AND/OR) were utilized to refine the search across the databases. A total of 8015 records were identified through the database searches, along with 409 additional records retrieved from other sources, including manual reference screening, WHO reports, and gray literature, yielding a total of 8424 records. After removing duplicates (*n* = 2880), 5554 records were screened on the basis of their titles and abstracts. Studies were included if they addressed ETEC epidemiology, virulence mechanisms, colonization factors, genomic diversity surveillance, or vaccine development. Articles not directly related to ETEC, those lacking relevant data, or those published in languages other than English were excluded. Following the screening process, 654 text articles were assessed for eligibility; 541 were excluded because they did not meet the inclusion criteria, had insufficient data, or lacked full-text availability. Ultimately, 110 studies were included in the final analysis. Priority was given to peer-reviewed articles that integrate current knowledge of ETEC molecular biology, detail virulence mechanisms, and explore emerging strategies for infection control, including vaccine development and One Health perspectives. The flow of this process is illustrated in the accompanying diagram (Supplementary Figure S1), which outlines how records were identified, screened, and assessed for eligibility, leading to the final set of included studies.

This review provides a comprehensive analysis of the global burden of ETEC, highlighting the structural and epidemiological diversity of its CFAs, the variability of its toxins and the latest advances in vaccine development within a One Health framework. We emphasize the antigenic variation observed in strains from both humans and animals and its implications for the design of multivalent vaccines. Despite advancements in molecular characterization and vaccine technology, significant knowledge gaps remain, including limited surveillance among adult and elderly populations, an incomplete understanding of regional strain diversity, and insufficient insights into cross-protective immunity among CFAs. Additionally, challenges in achieving broad and durable protection against infections remain. Addressing these gaps is crucial for the effective development of global preventive strategies. We have included a summary of the key epidemiological indicators to provide a clear overview of the global burden of ETEC infection (Table 1). This table highlights the prevalence, mortality rates, age distribution, and major risk factors in endemic regions.

### ETEC: An Intestinal Pathogen Found Worldwide

ETEC is a leading cause of acute diarrheal disease and mainly affects vulnerable children younger than five years of age in low- and middle-income countries. Several susceptible populations in developed countries can also be affected by intestinal infections caused by this pathogen. The high prevalence of ETEC cases, as described in several epidemiological studies, together with occasional institutional healthcare-associated outbreaks underscore the importance of adopting a comprehensive approach to address this issue. A One Health approach is critical, as it addresses human, animal and environmental health as an interconnected and dynamic system. Diarrheal diseases associated with ETEC infection have been reported predominantly in low- and middle-income countries (LMICs), as defined by the World Bank, where ETEC accounts for moderate to severe cases of diarrhea in children under five years of age, particularly in sub-Saharan Africa and South Asia, representing a substantial public health burden [28,29,30,31,32]. Nevertheless, sporadic cases and small outbreaks have also been reported in high-income regions [33] and are most frequently associated with international travel or foodborne exposure rather than with ongoing endemic transmission. These reports do not indicate a comparable disease burden between regions although ETEC remains globally relevant in our increasingly interconnected world [13,34,35]. Notably, the complex dynamics of ETEC transmission highlight the need for coordinated surveillance, prevention and control strategies that extend beyond individual healthcare settings. In this review, we discuss this coordination within a One Health-informed framework that emphasizes the role of environmental exposure, water quality, sanitation, and food. This approach does not imply zoonotic transmission; rather, it provides a structured framework for integrating public health, environmental, and policy considerations into coordinated surveillance and prevention strategies for ETEC. In 2006, an outbreak of 200 cases was reported in Denmark. In Norway, 110 cases were documented between 2012 and 2016. Moreover, 131 outbreaks occurred in Japan between 1966 and 2009 [36,37]. According to the literature, published data on the epidemiology of intestinal diseases caused by ETEC suggest that this globally distributed pathogen is not limited to impoverished regions but instead represents a transnational challenge requiring systematic and coordinated participation from diverse sectors in a comprehensive collaboration to address ETEC outbreaks.

**Figure 1:**
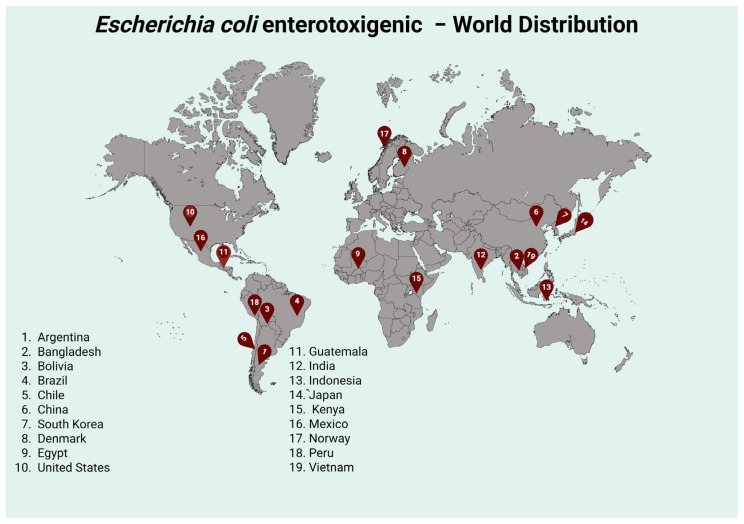
Global geographic distribution of ETEC. The countries highlighted on this map represent examples of reported epidemiological contexts in which ETEC has been documented on the basis of the available published data and representative studies from endemic regions. This selection does not imply the absence of ETEC in nonhighlighted countries, as this pathogen has a broad global distribution. Rather, the map is intended to emphasize the diverse settings impacted by ETEC and to underscore the continued need for effective vaccine development and implementation strategies worldwide. Created using BioRender, Rodriguez, R. (2025) https://BioRender.com/b2nui7z accesses on 5 July 2025.

The problem of intestinal infections requires urgent action in several Latin American countries (including Argentina, Bolivia, Brazil, Chile, Guatemala, and Mexico), which have reported clinically significant outbreaks of ETEC [22,36,37,38]. In 2000, an outbreak of 1521 cases was reported in Chalco Valley in the state of México, Mexico; of these cases, 62.2% were identified as ETEC, indicating a significant health burden. Several epidemiological studies from Mexico have indicated an ETEC incidence rate of approximately 33%, making Mexico among the countries with the highest incidence in Latin America [39]. These statistics highlight the urgent need for specialized epidemiological research and specific control measures tailored to the country’s condition. The genetic diversity of ETEC, the circulation of multiple serotypes (including O6, O27, O148, O159, and O169), and the increasing resistance of ETEC to several antimicrobial families in many countries (including Vietnam, India, and Kenya) limit the effectiveness of treatments to eradicate this pathogen [35,40,41]. In this context, the development and implementation of multivalent vaccine formulations designed to provide broad protection against geographically diverse ETEC strains is crucial. Analysis of intestinal infections from a One Health perspective can facilitate the integration of human epidemiological surveillance and environmental monitoring and animal–human interface assessments to coordinate clinical and preventive responses. ETEC can be transmitted through contaminated water, food, and fecal–oral encounters. Given its disproportionate effect on young children, establishing and maintaining coordinated environmental monitoring systems is essential. These systems are important for increasing access to sanitation, educating communities, and implementing effective immunization strategies. These joint efforts will help reduce the global burden of this disease, aid in the anticipation of emerging outbreaks, and promote health equity. The global distribution of ETEC is shown in Figure 1.

ETEC poses a significant threat to public health and affects the most vulnerable communities worldwide. A more effective alternative for addressing challenges related to intestinal infections is the comprehensive One Health approach. This strategy focuses on developing sustainable and efficient interventions that consider local ecological, social, and geographic contexts, particularly with respect to surveillance, prioritization, and public health deployment, to counteract the direct health effects of ETEC infection.

## 2. Antigenic Heterogeneity and Colonization Complexity in ETEC

Among the various types of intestinal pathogens, ETEC is noteworthy for its immense versatility and broad repertoire of CFAs, which have adapted to infect humans. The main CFAs include fimbrial adhesins, nonfimbrial adhesins, and flagella, all of which enable bacteria to adhere to and colonize intestinal cells. In addition, ETEC produces two toxins, heat-labile toxin (LT) and heat-stable toxin (ST), which disrupt intestinal fluid homeostasis through the activation of cyclic AMP and cyclic GMP signaling pathways, respectively, and play important roles in the development of diarrheal diseases [42,43,44,45]. Following antigen exposure, particularly after mucosal immunization, ETEC antigens such as CFAs and detoxified toxin derivatives are captured by intestinal dendritic cells, which then migrate to mesenteric lymph nodes and initiate antigen presentation to CD4+ T cells. This interaction promotes the differentiation of these cells into Th2 and Th17 phenotypes, both of which are essential for effective mucosal immunity. Th2-associated cytokines facilitate B-cell activation and immunoglobulin class switching, resulting in the production of antigen-specific secretory IgA (sIgA) at the intestinal surface. Secretory IgA plays a central protective role by inhibiting bacterial adhesion to epithelial receptors and neutralizing enterotoxins. In parallel, memory B cells and long-lived plasma cells contribute to sustained immune responses. While LTs are highly immunogenic and can induce both systemic and mucosal immune responses, STs exhibit low intrinsic immunogenicity and often require molecular modification or conjugation strategies to increase immune recognition. These immunological mechanisms are critical for the rational development of vaccine candidates [21,46,47,48]. However, unlike those of other pathogens, the mechanisms underlying ETEC pathogenicity have not yet been extensively described. This lack of detail can be attributed to the high variability of the structures involved in host colonization. More than 20 CFAs have been identified in several ETEC strains and are involved in infectious processes in animals and humans. CFAs are expressed in diverse combinations, promoting the wide heterogeneity of ETEC strains and dictating the presence of specific ligands in the host. ETEC CFAs/Coli surface antigens (CSs) primarily bind to specific glycolipid and glycoprotein receptors present in the brush border of the small intestine. Many CFAs recognize glycosphingolipids; for example, CFA/I binds to several neutral glycosphingolipids, such as glucosylceramide and glucosylceramide, as well as other more complex derivatives. CS6 specifically recognizes the glycosphingolipid sulfatide, which has been detected in the small intestine of humans and other susceptible species [49]. Similarly, the colonization factor CS30 binds to sulfatide in human and porcine intestines, reinforcing the role of this lipid as a key receptor for certain ETEC variants [50]. Other CFAs, such as components of CFA/II (e.g., CS3), bind to specific brush border membrane glycoproteins, as demonstrated by the inhibition of glycopeptide adhesion and the characterization of low-molecular-weight receptor proteins in human intestinal cells. In summary, carbohydrate-based receptors are responsible for the host and tissue specificity of ETEC colonization [44,51]. This variability highlights important challenges for clinical approaches to treat ETEC infections [44,45,52]; the clinical implications of this variability are significant and compelling, underscoring the need for comprehensive, targeted mechanistic research. A clearer understanding of the diversity of toxin expression patterns and host–pathogen interactions involving CFAs is necessary for the development of effective preventive and therapeutic strategies.

### 2.1. Mechanisms of ETEC Colonization

**Figure 2:**
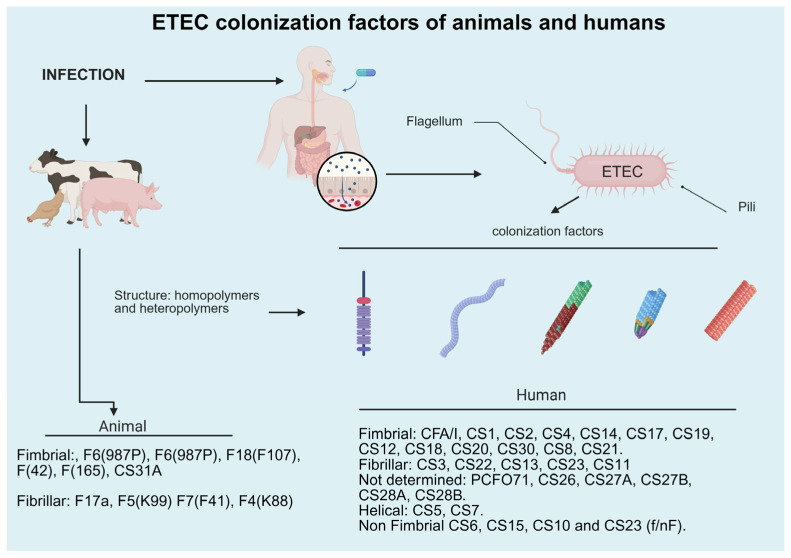
Diversity of the ETEC CFAs that promote pathogen colonization in both animals and humans. ETEC has various factors that facilitate its colonization in both humans and animals. Twenty-nine of these CFAs have been detected in humans, whereas nine have been detected in animals. These CFAs can occur in different forms, including fibrillar, fimbrial, and nonfimbrial configurations. Additionally, they can adopt either homopolymeric or heteropolymeric structures, as illustrated in this figure. The importance of these CFAs lies in the ability of ETEC to use them to colonize the small intestine after a person consumes contaminated food. F, fimbrial; f, fibrillar; nF, nonfimbrial; H, helical; and n.d., not determined. Intriguingly, the sequences and functions of CS10 and CS11 remain unknown [45]. This figure depicts important structures, such as flagella and pili, which are filamentous surface appendages involved in bacterial adhesion to host epithelial cells and play critical roles in intestinal colonization; these structures are considered accessory structures that are involved mainly in bacterial motility. Created using BioRender. Rodriguez, R. (2025) https://BioRender.com/f601yce accesses on 5 July 2025.

As previously described, the most well-studied CFAs involved in several classical colonization mechanisms include CFA/I, CFAs/II (CS1, CS2, and CS3), and CFA/IV (CS4 to CS6). Other studies have identified additional fimbriae (CS14, CS17, CS21, and CS26), along with PCF071 and EtpA, thereby paving the way for further research and a better understanding of this field [11,44,53,54,55]. Studies have shown that ETEC strains can produce multiple CFAs, either individually or in combination. However, not all fimbriae have been fully characterized, as their expression depends on various factors, such as environmental conditions, immunotherapy, osmolarity, pH, and temperature. Despite limited research, the results described to date provide interesting opportunities for the development of more sensitive detection methods that will improve our understanding of ETEC. Moreover, the frequent coexistence of multiple CFAs within a single strain underscores the need for multivalent or modular vaccine strategies that can address antigenic heterogeneity. Although recent research reports are lacking, the diversity described to date highlights the importance of integrating genomic, phenotypic and ecological data to refine both surveillance and rational vaccine development efforts. Future research aimed at identifying some of these fimbriae may inspire the next generation of microbiologists. Distinguishing classical fimbrial CFAs from accessory surface structures involved in motility or host interactions, such as flagella, which are not classified as canonical ETEC CFAs, is important (Figure 2). Unlike the adhesion structures found in commensal *E. coli* strains, the CFAs of ETEC are virulent. Associated fimbrial structures are frequently encoded on plasmids that also carry enterotoxin genes. Their expression is tightly regulated by environmental signals such as temperature, osmolarity, and bile salts encountered in the small intestine. Importantly, CFAs exhibit specific receptor binding properties that facilitate targeted epithelial attachment and are directly linked to toxin-mediated diarrheal disease. In contrast, adhesion structures in commensal *E. coli* are typically chromosomally encoded, contribute to stable intestinal colonization and lack an association with enterotoxin production or acute inflammatory responses [51,56,57].

The wide variety of CFAs in different ETEC strains indicates that this bacterium readily adapts to its environment, with adaptive advantages conferred by acquiring genetic material through horizontal transfer and integrating it into its genomic or plasmid DNA. Plasmid DNA coexists independently of chromosomal DNA in bacteria and plays a crucial role in rapid adaptation and evolution by facilitating the transfer of genetic material between bacteria. This adaptability is a key strategy for ETEC survival that endows this pathogen with a significant advantage in diverse ecological environments, including those inhabited by humans and animals. The high variability of this pathogen offers an important adaptive advantage from an evolutionary perspective but represents a significant and urgent global challenge for the development of viable vaccines. This compilation of data includes some strategic findings for the development of possible vaccines from CFAs in ETEC strains. This information raises the following relevant question: How can we generate an effective immune response against molecular targets that differ between strains or vary by geographic region? The efficacy of the immune response must be contextualized as a pivotal event in the intimate relationship between bacteria and the host. Unlike stable toxins (LTs or STs), CFAs have significant antigenic and structural diversity. This variability makes the use of CFAs as universal antigens in multivalent vaccines challenging [58,59].

### 2.2. Incomplete Mapping: Limits in Surveillance and Characterization Using State-of-the-Art Technology

Despite significant advances in sequencing technologies and emerging fields such as omics, a clear consensus on the classification and distribution of CFAs in ETEC has yet to be reached. Genomic studies have shown that many CFAs traditionally classified as “classic” are challenging to detect in clinical isolates from regions with ETEC outbreaks. In contrast, adhesins embedded in different CFAs that are considered nonclassical are more prevalent and highly variable [52,60,61]. This discrepancy highlights the gap between ongoing research and the mechanisms by which external factors influence CFA expression. Several factors may contribute to this situation, including the limitations of conventional phenotypic assays, poorly characterized outbreaks among travelers, and a general lack of research on cases arising in low-income countries, such as Mexico. Therefore, a new approach is needed, as this pathotype is not routinely examined in cases of childhood diarrhea. Comparative genomics presents an alternative solution. Multigenomic analysis of ETEC strains enables researchers to identify clusters of fimbriae and adhesins and to conduct phylogenetic analyses that reveal significant epidemiological or immunological relevance compared with classical CFAs. Increasing evidence indicates that CFAs should not be viewed solely as isolated multigenic adhesive fimbriae but rather as components of a coordinated colonization system that interact with additional virulence determinants. Recognizing this functional complexity may inform the rational design of vaccine strategies to achieve broader coverage despite the high intraspecific variability of ETEC. Recent evidence suggests that CFAs may function as components of multifactorial colonization systems involving coordinated interactions with additional virulence determinants [62].

Postgenomic mapping using next-generation sequencing (NGS) technologies is a computational biology tool that has recently transformed our understanding of how pathogenic bacteria, such as ETEC, cause intestinal diseases. These studies are not limited to superficial analyses but provide comprehensive genetic data, including data at the nucleotide level. Intestinal pathogens such as ETEC colonize mainly humans and are associated with high antigenic variability and adaptability to different environments. Genomic research has helped elucidate the complexities of different CFAs and their relationships with toxins. Other studies on ETEC have focused on characterizing several serotypes, providing limited information on the diversity of this pathogen. Dynamic genetic repertoires, virulence clusters, and other mobile genetic elements are adaptive advantages that contribute to the pathogenicity of ETEC. In this review, the relevance of ETEC is highlighted from a dynamic perspective by examining the concepts that characterize its adaptability and evolutionary flexibility. Approaching ETEC through an evolutionary process as a complex and dynamic system has been increasingly supported by comparative genomic studies using next-generation sequencing technologies [61].

Comparative studies of the genomes of ETEC strains from human, animal, and environmental sources have identified conserved genetic traits that are essential for the biological functions of this pathogen. These genetic signatures function as important indicators, providing valuable insights into the development of effective and representative vaccines [15]. Using this approach, researchers can identify genes that regulate key functions, such as the adhesion process within different CFAs and the production of toxins (both thermolabile and thermostable), in ETEC pathogenesis. In addition, it is important to emphasize the relevance of the different genetic elements that are not merely auxiliary but instead confer critical adaptive advantages that allow this pathogen to thrive and spread to diverse hosts and ecosystems [36]. In this context, comparative genomics plays a crucial role in identifying and prioritizing candidate vaccines on the basis of several criteria, including evolutionary conservation, gene expression levels, the potential to induce an immune response, and epidemiological significance, via a strategic rather than an arbitrary approach [63]. A new generation of innovative vaccines has been developed on the basis of recent advances in sequencing technologies, reverse vaccinology platforms, and computational structural modeling [21]. These vaccines have been generated through rational design strategies that incorporate multiple epitopes and selected antigens identified through genomic and computational screening, followed by experimental validation and controlled preclinical and clinical evaluations. These systems focus on producing broad protective immunity or cross-reactivity by utilizing conserved epitopes from various CFAs, adhesins, and inactivated toxins [21,64]. This strategy considers the genetic diversity of ETEC, enabling the development of novel, potentially specific vaccines that shift away from a universal model toward formulations suited to particular epidemiological contexts.

The ability to integrate advances from the One Health approach extends beyond simple categorization and requires a clear understanding of the complex ecological connections involved. Genetic diversity in ETEC strains is a mechanism of adaptation and evolution that significantly and directly affects their movement and cycling between human and animal populations, including in contaminated environments such as water bodies, food irrigated with sewage, and poorly handled meat. In this context, phylogenomic analysis (especially in agricultural or semiurban areas) can enhance integrated surveillance by identifying environmental and host-associated sources and providing a clearer understanding of transmission dynamics that may not involve routine clinical observations [65]. The development of effective and sustainable control strategies is essential for reducing the impact of pathogens such as ETEC. These new techniques generally share the common goal of developing personalized, long-lasting, and effective vaccines that contribute to human well-being. Comparative genomics plays a crucial role in this process by identifying the parts of a pathogen that need to be targeted and providing important insights into its ability to cause disease, its origins, and its mode of transmission [15]. In a global context, this information must be contextualized, as individual health, rural health, and environmental conservation are closely related [6,66]. This approach is gaining increasing attention in academia and urgently needs to be adopted worldwide to address the impact of ETEC on intestinal infections. A comparative overview of the main CFAs of ETEC, including their classification, distribution, toxin profiles, associations, and significance for vaccine production, is presented in Table 2.

### 2.3. Ecological Diversity Beyond the Human Gut

As previously described, ETEC is an intestinal pathogen that affects humans, cattle, and pigs. ETEC has also been isolated from companion animals and from contaminated food, water, plants, and other environmental sources. The diverse sources of ETEC are associated with the presence of virulence factors that are similar to or highly correlated with the CFAs described in human ETEC strains. These findings indicate that gene transfer and mixing occur, thereby favoring the heterogeneous distribution of ETEC strains. This dynamic process contributes to the adaptability of ETEC, as well as to the challenges that have arisen for its control [40,67]. Therefore, we must emphasize that these findings highlight the critical importance of adopting a One Health approach.

**Figure 3:**
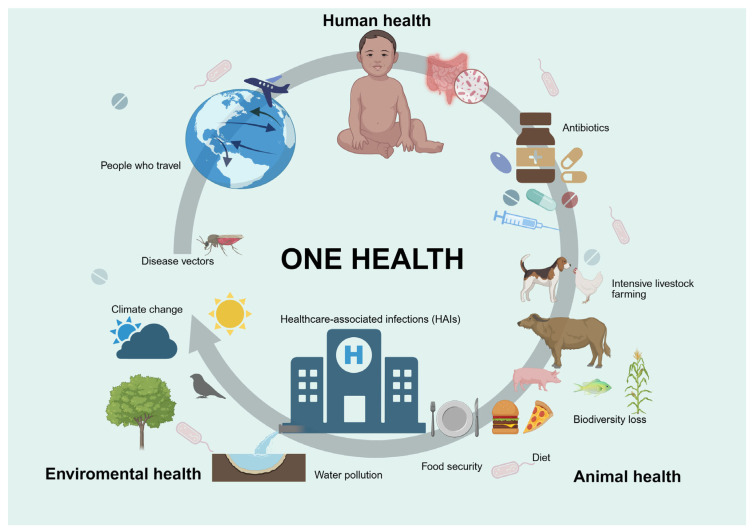
One Health Perspective. Diarrheal diseases caused by ETEC strains pose risks to human and animal health, primarily in low-income countries. In this context, ETEC pathogenesis involves transmission and includes transmission routes, environmental persistence and the presence of reservoirs in domestic animals, all of which contribute to the deterioration of human health. Conversely, a One Health strategy that combines human medical therapy, animal health management and environmental cleanup is necessary to effectively treat these diseases. The development of new candidate vaccines against ETEC will significantly reduce the disease burden and resistance of ETEC strains to several families of antibiotics. Developed with BioRender. Rodríguez, R. (2025) https://BioRender.com/0grzebx accesses on 5 July 2025.

The One Health strategy focuses on integrating information through an interconnected network of human, animal, and environmental health to better address the epidemiology of ETEC. This approach provides us with detailed insights into the emergence of virulence factors within specific ecological contexts and their potential dissemination under environmental and host-associated selective pressures [68]. This approach is an integral and systematic strategy that is essential for combating pathogens such as ETEC. A schematic of ETEC from a One Health perspective is shown in Figure 3. In summary, several virulence factors pose technical challenges and conceptual and practical barriers to ETEC treatment. Therefore, a re-evaluation of current methods for developing effective vaccines is needed. New adaptive solutions that integrate genomic data and are appropriate for specific ecological contexts and geographic areas must be developed to move beyond the one-size-fits-all approach.

## 3. Current Status of Vaccine Strategies Against ETEC Strains

The development of effective ETEC vaccines remains challenging because of the high genetic and antigenic diversity of the circulating strains. The prevention of millions of cases of childhood diarrhea caused by this pathogen is a clear and urgent goal but daunting task. When it seems that the scientific community is on the verge of a breakthrough, new challenges arise that impede the development of optimal vaccines. One of the most significant obstacles is the diversity of ETEC CFAs and toxin profiles, which has proven to be a significant and persistent barrier, limiting the efficacy and cross-protective coverage of the candidate vaccines developed to date. Nevertheless, a solution to this problem will have a major impact and provide hope, as it offers a glimpse into a future focused on a significant reduction in ETEC cases [69,70].

### 3.1. The Traditional Method: Multivalent Vaccines Targeting CFAs

The first vaccines targeted the most common CFAs that were known at that time. A recent phase 1 clinical trial assessed the safety and efficacy of a multivalent vaccine in producing immunological responses in 129 participants. This vaccine was a multivalent, inactivated oral vaccine that could be administered either alone or in combination with the adjuvant dmLT, a double-mutant heat-labile toxin. The vaccine was composed of four recombinant strains that were deliberately inactivated to create four CFAs: CFA/I, CS3, CS5, and CS6. Additionally, the vaccine contained a toxoid linked to the B subunit of the LT. The results demonstrated that the vaccine was safe and stable; moreover, it produced a strong immune response. Additionally, 83% of vaccinated individuals generated a mucosal IgA response to the primary antigens. In this context, the dmLT adjuvant played a crucial and promising role by significantly increasing the immune response, providing new possibilities for further improvement [71]. A phase 2B, randomized, double-masked, placebo-controlled clinical trial assessed the safety and immunological effectiveness of the ETVAX vaccine, which comprises four inactivated ETEC strains and includes the B subunit of the LT. This trial included 749 travelers or tourists from Finland who visited Benin, a West African nation. In this trial, the immunogenicity of the vaccine was assessed by quantifying the titers of IgA and IgG antibodies against LTb and lipopolysaccharide O78. The results revealed that the ETVAX vaccine was safe and caused a strong immunological response; that is, an increase in the level of IgA against LTb was observed. Eighty-one percent of those who were vaccinated responded to LTb, and 69% responded to O78. In total, 93% of the subjects responded to at least one of the tested antigens, and no significant adverse effects were recorded. These results indicate that the ETVAX vaccine could be viable and efficacious in reducing ETEC diarrhea among tourists [72]. The development of new candidate vaccines against ETEC is a critical component of the One Health approach for addressing diarrheal diseases. Several ETEC vaccine candidates are currently in different stages of clinical development (Table 3).

### 3.2. The Achilles’ Heel: Variability and Evasion of Immune Responses

Limited vaccine coverage due to the ability of ETEC to evade the immune system is another serious concern, which suggests that ETEC has developed new mechanisms, such as altering or replacing its CFAs with mobile genetic elements. Consequently, ETEC can evade the immunity generated by previous infections or vaccinations. In essence, traditional APC-based vaccines can provide long-lasting immunity but only against a small subset of this vastly diverse pathogen, similar to the behavior of the influenza virus from an evolutionary perspective [43,80]. In a constantly changing environment, the target of the immune system also continually shifts. However, unlike that of influenza viruses (which are monitored worldwide), the genetic surveillance of ETEC is generally in its early stages or entirely nonexistent [81]. These networks tend to prioritize monitoring travelers over local populations, which underscores the urgent need for better vaccine adaptation.

### 3.3. Evaluation of Vaccine Strategies: Toxins and Adjuvants

Effective vaccine that target the activity of the toxins produced by ETEC strains, specifically the two types of enterotoxins (STs and LTs), is a significant challenge. STs are small molecules that do not induce a significant immune response and are structurally similar to specific human peptides, such as guanylin and uroguanylin, which can cause issues with cross-reactivity. In contrast, LT has been used as an immunogen and an adjuvant in several vaccine formulations, with inconsistent results reported. Some studies have suggested combining ETEC antigens with antigens from other pathogens as an alternative strategy to increase immunogenic activity. This approach has been used in multivalent vaccines designed to prevent childhood diarrhea caused by various pathogens, such as Shigella, Campylobacter, and rotavirus [78]. The potential of multivalent vaccines to transform ETEC vaccination strategies is considerable. These strategies are promising options for improving public health, as they lead to the generation of a significant immune response against ETEC, particularly if the incorporated antigens do not match the prevalent strains in each region or healthcare setting.

### 3.4. Ethical and Practical Dilemmas in Implementation

Several ETEC vaccine candidates currently under development can reduce the disease burden in low- and middle-income countries where ETEC remains endemic and disproportionately affects young children. These efforts are driven primarily by the need to prevent severe diarrheal disease and associated mortality in resource-limited settings.

In this context, travelers from high-income countries represent a secondary target population, as vaccine strategies developed for endemic regions may also confer protection against travel-associated ETEC, which aligns with global health priorities rather than the exclusive protection of populations in wealthy countries. The latter approach poses both ethical and practical challenges. From an ethical standpoint, these vaccines are designed for individuals at a lower risk, such as travelers. Moreover, the most vulnerable populations, such as children in developing countries, remain unprotected and urgently require assistance [63]. The administration of oral vaccines poses logistical difficulties and challenges related to storage and high manufacturing costs, making their implementation difficult in populations in rural or impoverished areas [3].

## 4. Innovations in Vaccine Technology: A New Generation to Address the Antigenic Diversity of ETEC

Examination of the extensive complexity of ETEC virulence from both academic and strategic perspectives is essential for determining its intrinsic properties. This intestinal pathogen is characterized by a broad repertoire of virulence traits that contribute to its colonization of the host, representing a considerable obstacle to its prevention via a traditional vaccination approach. Thus, the search for alternative methods to generate new vaccines has been ongoing and has resulted in a notable transformation in scientific research methodology. Rather than seeking a single, functional response, recent studies have focused on examining more flexible and responsive immunological frameworks that include real-time data. These innovative approaches focus on generating knowledge to transform vaccine development by incorporating new genetic engineering and bioinformatics technologies. Recent advances in alternative perspectives have facilitated a comprehensive assessment of the design and implementation of next-generation vaccines [5,61,69,82,83].

### 4.1. Reverse Vaccinology: From Genomic Analysis to Rational Antigen Selection

Reverse vaccinology is a genome-based approach that facilitates the systematic identification of candidate antigens from pathogen genome sequences, particularly in organisms with substantial antigenic diversity, such as ETEC. Rather than relying solely on a phenotypic screen, this strategy enables the comparative analysis of genetically conserved proteins across diverse clinical isolates. However, the expression, surface accessibility, functional relevance and immunogenicity of the identified candidates must be evaluated before they can be considered viable vaccine targets [84]. In this context, reverse vaccinology for ETEC can contribute to the identification of nonclassical adhesins such as EtpA, EtpA, and YghJ, which are more broadly conserved than many classical CFAs are. Although these antigens represent promising complementary targets, their variable expression and regulation underscore the need for careful functional validation with multicomponent vaccine strategies [60,85,86].

### 4.2. Experimental Validation and Immunological Characterization of Selected Antigens

Reverse vaccinology has emerged as a pivotal step in the development of next-generation vaccines for pathogens with high antigenic diversity such as ETEC. Reverse vaccinology uses sequencing technologies to identify protein sequences that are genetically conserved across diverse clinical isolates. These candidates are subsequently evaluated for surface expression and exposure and immunogenic potential before being considered molecular targets in vaccine research and development [63]. In this specific context, several nonclassical adhesins (including EatA, EtpA, and YghJ) that also contribute to the adhesion and colonization of ETEC in its hosts have been identified. These nonclassical adhesins exhibit a high degree of conservation compared with traditional CFAs. Importantly, in addition to their roles in colonization and immune evasion, some of these proteins generate significant immune responses in animal models. These findings indicate their potential as broadly conserved components for the development of multiepitope vaccines [54,64].

### 4.3. Multiepitope Vaccines and Structural Immunogen Design

The generation of multiepitope vaccines is among the most promising strategies for developing vaccines that induce humoral and cellular responses to medically important pathogens. These innovative vaccines, which are characterized by the combination of peptides from various antigens (including CFAs, toxins, and adhesins), are designed with artificial structures and have shown great potential [87,88]. Multiepitope vaccines can incorporate peptides from ETEC toxins and conserved adhesins, allowing the simultaneous targeting of colonization and toxin-mediated pathogenic mechanisms. This approach not only expands the range of clinically relevant target strains in a specific area but also reduces the risk of missing relevant antigens, offering hope for future vaccine development. Artificial intelligence is revolutionizing vaccine design; its integration into the natural sciences has not only optimized the stabilization of vaccine structures and their solubility but has also significantly improved vaccine production. Innovations such as AlphaFold enable the rapid prediction of protein structures, which favors the most effective and efficient process for developing potential vaccines, significantly reducing the time needed to generate a vaccine [89].

### 4.4. mRNA Vaccines: Speed, Adaptability, and Potential

The synthesis of efficient mRNA vaccines against SARS-CoV-2 has stimulated interest in exploring this platform for infectious diseases beyond viral pathogens, including enteric bacteria such as ETEC [90]. The ability of these mRNA platforms to encode multiple antigens within a single construct theoretically offers flexibility in targeting heterogeneous bacterial virulence factors. Given the antigenic variability of ETEC, such modular design strategies may facilitate iterative antigen selection and optimization. However, the application of mRNA vaccines against enteric bacterial pathogens poses significant challenges. A key limitation involves the difficulty in inducing strong mucosal immunity, as most available mRNA platforms are parenterally administered and primarily elicit systemic immune responses (rather than the necessary intestinal IgA-mediated protection). Additionally, the selection of appropriate antigens is complicated by the high diversity and host specificity of ETEC colonization factors, which may limit cross-protective efficacy. Structural differences between viral and bacterial antigens (including the need to target surface-exposed adhesins and toxins) further complicate antigen design.

The high adaptability of pathogens such as ETEC is evolutionarily advantageous, indicating that the development of new vaccines must also adapt to the constantly evolving conditions in nature. Furthermore, mRNA vaccines do not require bacterial cultures or specific pathogens for their production. In this context, compared with traditional vaccine production methods, vaccine development is faster and involves minimal biological risk. Although mRNA-based vaccines against ETEC are advantageous, current evidence is mostly limited to preclinical models rather than human studies. While mRNA vaccines offer a new and effective approach to immunization, their potential for controlling ETEC should be viewed with caution, as significant challenges remain regarding their stability in mucosal environments and their large-scale implementation in endemic regions. These issues have not yet been fully addressed [83]. One of the key features of this cutting-edge technology is the ability to regulate the expression of several specific antigens in the epithelium, which is the primary site of ETEC colonization. This increases mucosal immunity, which is an essential step in combating intestinal infections caused by ETEC. Although few human clinical trials have investigated mRNA-based vaccines against ETEC, recent preclinical models have shown promising immune responses against pathogens such as Shigella, Salmonella, and *E. coli* [91,92]. The mRNA platform provides significant advantages and unique opportunities for developing vaccines against ETEC. For instance, mRNA constructs can be designed to encode combinations of conserved CFAs, derivatives of heat-labile toxins, or multiepitope antigens that represent various ETEC lineages. These strategies could enable the rapid evaluation of multivalent antigen combinations, which is essential for addressing the extensive genetic and antigenic diversity observed among ETEC populations.

### 4.5. Nanoparticle Vaccines: Targeted Antigen Delivery Strategies

The development of recombinant proteins formulated within nanoparticle-based systems has enabled better control of antigen presentation and targeted delivery strategies, including approaches that target components of the intestinal immune system, such as Peyer’s patches. In the context of ETEC, nanoparticle platforms may protect structurally complex antigens such as conformational CFAs during gastrointestinal transit. Some experimental formulations have been designed to increase stability under acidic conditions and promote localized immune responses after oral administration [83]. By incorporating multiple antigens into a single system, nanoparticle-based vaccines may facilitate multivalent presentation, which is particularly important for pathogens with high antigenic diversity, such as ETEC. However, their efficacy and durability in humans remain under evaluation. Certain formulations involving plant-derived biomolecules or biodegradable polymers as carrier systems are also being explored to improve scalability and reduce production costs in low- and medium-income settings [84].

### 4.6. Limitations, Challenges, and Ethical Considerations

The new technologies described in this review have significant transformative potential; however, specific challenges hinder their widespread implementation. One significant problem is the lack of infrastructure required for mRNA or nanoparticle production in different regions of the world. Despite significant progress, considerably more research on regulation (particularly in terms of ethical considerations and the development of regulatory frameworks for implementing innovative vaccines in the most vulnerable populations) is needed. Ensuring transparency and rigor in these procedures is essential. The complexity of such implementation could lead to limited product availability, thereby restricting access to preventive interventions such as vaccines among the populations most need it. New vaccines must be developed with a strong emphasis on equity, scalability, and local technology transfer from the earliest stages to prevent this paradox, which could significantly increase the reach and impact of vaccines [93,94].

Genomic tools, reverse vaccinology, and mRNA vaccines are emerging innovative technologies that offer viable alternatives for pathogen surveillance and control. However, structural barriers and challenges persist in the implementation of these technologies worldwide from a One Health perspective. These difficulties include technical and ethical issues that require thoughtful and fair assessment [87]. However, these vast opportunities are often overshadowed by tangible factors such as a lack of the infrastructure, knowledge, and resources necessary for effective implementation. Instead, research often focuses on privileged communities, creating a worrisome disparity between technological advances and their practical use in real-world settings.

## 5. ETEC from a One Health Perspective: Understanding the Causes of Diarrhea Other than Contaminated Water

Throughout history, the effects of ETEC-induced diarrhea have been addressed exclusively from a clinical perspective related to rural hospitals, contaminated food outbreaks, and affected children, particularly in developing countries. However, several studies have revealed additional details of an ecological network in which ETEC persistence is maintained, its transmission is favored, and its genetic repertoire is enriched through horizontal gene transfer mechanisms. On the basis of these data, focusing solely on elucidating how ETEC affects humans is both limiting and negligible, as it does not address all the elements related to diarrheal diseases. Therefore, addressing the effect of ETEC from a more comprehensive perspective is important [95]. As a distinct conceptual framework, the One Health approach promotes the integration of human, animal, and environmental health as a single perspective, thereby emphasizing the close connections among these three areas [29]. In this context, ETEC is considered an ecological factor and pathogen within an integrated dynamic system. This complexity is both fascinating and challenging, inviting more detailed exploration and analysis.

### 5.1. Animals as Reservoirs: The Unwitting Hosts of the Transmission Cycle

Although most studies on ETEC have focused on the human population, several farm animals (including cattle and pigs) and wild animals (including birds) can act as asymptomatic reservoirs [45]. Recent studies have demonstrated that some virulence-associated genes may share certain genetic elements between animal and human ETEC strains [96]. However, most colonization factors and fimbrial adhesins are host specific and adapt to distinct intestinal receptors. These findings highlight potential ecological overlap; however, they do not provide evidence of direct zoonotic transmission. Further studies are needed to clarify the epidemiological relationships among the human, animal and environmental sources of ETEC and their contributions to the disease burden in children. Comparative genomic analyses indicate that although human and animal ETEC strains may share certain virulence-associated gene families, they generally segregate into host-adapted lineages characterized by distinct colonization factor repertoires and regulatory patterns. Other studies have indicated that livestock-associated ETEC strains commonly express fimbrial adhesins adapted to specific animal intestinal receptors, whereas human ETEC strains display CFAs that are optimized for the human small intestine. The specificity of ETEC CFAs limits their transmission between species, thus reinforcing fecal-oral human-to-human transmission as the primary epidemiological route (rather than direct zoonotic transmission). Within a One Health framework, animal-associated ETEC strains primarily play a role in integrated surveillance. This process includes monitoring the diversity of virulence genes and the dynamics of antimicrobial resistance rather than serving as evidence of direct zoonotic transmission or being considered targets for cross-species vaccine development [21,97,98].

### 5.2. The Essential Importance of One Health in Children’s Well-Being: An Integrated Approach for Sustainable Solutions

**Figure 4:**
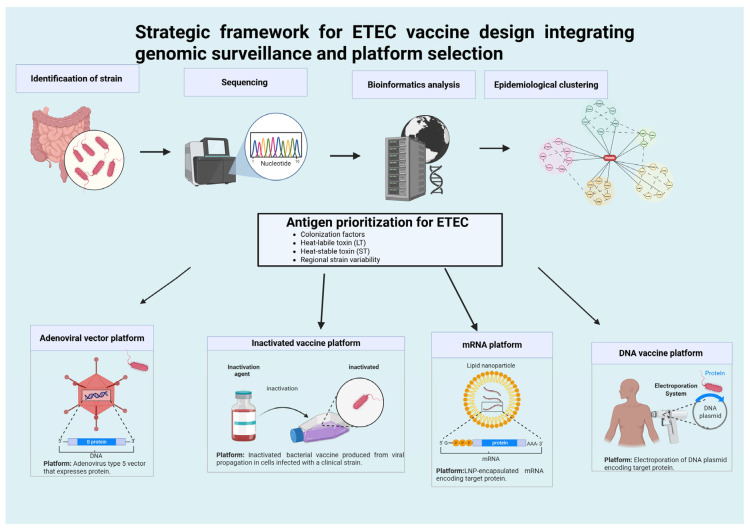
Conceptual Framework for ETEC Vaccine Development: Integration of Genomic Surveillance and Platform Selection. This diagram outlines a stepwise workflow for identifying and characterizing ETEC at the genomic level. It proceeds to the bioinformatic prioritization of key antigens, including colonization factors and enterotoxins. On the basis of this selection process, various vaccine platforms (including inactivated vaccines, mRNA vaccines, DNA vaccines, and adenoviral vector vaccines) are evaluated. Although these platforms offer distinct advantages in antigen design and development, their application to enteric pathogens presents challenges related to mucosal immunity, antigenic diversity, and implementation in resource-limited settings. This framework highlights the importance of integrating genomic and epidemiological data to inform context-specific vaccine strategies. Created with BioRender. Rodriguez, R. (2025) https://BioRender.com/spulnxu accesses on 5 July 2025.

Effectively addressing the concerns surrounding ETEC infection requires innovative methods that promote the integration of public health, veterinary medicine, and microbial ecology. New multiomics and bioinformatics technologies support the development of integrated genomic surveillance systems to track ETEC outbreaks in humans, as well as outbreaks of other pathogens affecting animals and the environment. Next-generation sequencing technologies and comparative genome analyses are two tools that contribute to elucidating the behavior of outbreaks associated with nosocomial pathogens [99,100]. The importance of implementing biosecurity measures in animal production to limit the spread of virulent and multidrug-resistant strains must be emphasized. Moreover, veterinary vaccines for multiple species that reduce the enteric burden of animals and act as ecological barriers to the transmission of infections must be developed. Additionally, equal access to health services and clean water infrastructure must be promoted, not as a postinfection treatment but as a fundamental tool for preventing various infections [10]. For this approach to be practical, a shift in thinking is needed to completely transform traditional vaccination into an essential tool within a comprehensive and environmentally sensitive health care system. The One Health perspective is a holistic approach that supports the well-being of children and prevents various diseases. It is a flexible and accessible vaccination system that can respond rapidly once the microorganism is identified. This methodology enables the selection of the most suitable strategies according to the specific needs and conditions of the area, as illustrated in Figure 4.

## 6. Challenges and Strategies for Sustainable ETEC Vaccine Implementation in Low- and Middle-Income Countries

The development of an effective ETEC vaccine is only part of the challenge; the real test lies in ensuring that the vaccine reaches those who genuinely need it and that its use is effective, fair, and sustainable. In developing countries such as Mexico, the burden of diarrheal diseases is particularly heavy in rural and disadvantaged areas, and the causative agents of these diseases are often not routinely identified [30]. Thus, the implementation of strategies to combat ETEC infections in other regions cannot be replicated in the same manner. Instead, these models must be adapted to the local context and consider the distinct histories and social realities specific to these communities. Such an approach is vital for immediately addressing the current strengths and key weaknesses.

### 6.1. Mexico as an Illustrative Case Within the Global ETEC Burden

Mexico is an illustrative example of a country marked by a unique mix of challenges and opportunities. On the one hand, inequality restricts access to essential services such as water, healthcare, and sanitation in Mexico, especially in rural and indigenous communities, as reported by CONAPO in 2020 [101]. On the other hand, the growing biotechnology infrastructure and research centers in Mexico have the potential to drive significant advances in fields such as biomedicine, recombinant vaccines, and multimolecular platforms, suggesting a promising future. A strategy that combines state-of-the-art research with practical applications is essential to maximize the benefits of this duality, similar to other endemic settings. Pilot vaccination programs could be established in certain regions, linking immunization campaigns to initiatives focused on environmental health, community education, and child nutrition. This approach involves the community in planning and monitoring vaccination programs [102,103].

### 6.2. Importance of Community Approval and Public Health Laws

A vaccine, regardless of its stage of development, will not be effective unless it is widely accepted and used. For successful implementation, communities must recognize, adopt, and proactively seek vaccines. In Mexico and other developing countries, vaccine implementation must be supported by communication strategies that respect local cultures and are developed in cooperation with community health promoters [104]. These promoters play a vital role in integrating traditional knowledge into the development of vaccination strategies. Furthermore, vaccination programs must be aligned with existing health systems to prevent duplication of effort and foster trust in health institutions. The principles of transparency, accountability, and high-quality service delivery are the foundations of quality healthcare. Public–private partnerships, collaborations between universities and local governments, and international supply chains are essential for the distribution, evaluation, and confirmation of the efficacy and financial viability of a vaccine.

### 6.3. Technological Accessibility: A New Perspective for Some or a Solution for All?

Despite these challenges, technologies such as mRNA vaccines, nanoparticles, and reverse vaccinology are emerging as tools to overcome these issues. However, these next-generation technologies also pose challenges for implementation in resource-limited countries. Therefore, realistic and viable strategies must employ low-cost, modular platforms that can be locally scaled, such as those based on recombinant proteins or attenuated bacteria, the effectiveness of which has already been indicated in diverse contexts [105,106].

## 7. Conclusions

Future research should focus on the development of an ETEC vaccine, as the ongoing challenges posed by ETEC require that research efforts remain evidence-based and aligned with its known epidemiological impact. ETEC is associated with watery diarrhea, dehydration and impaired growth; moreover, it contributes to substantial morbidity in endemic regions. These health outcomes occur within broader contexts that include socioeconomic disparities, environmental exposure risks and limitations in healthcare infrastructure. In this context, vaccinology can be considered a complementary and adaptive strategy (rather than a standalone solution aimed at addressing antigenic variability and transmission complexity) [68].

An important conclusion of this review is that the diversity of ETEC virulence factors is not random; rather, it is influenced by selective pressures that drive ongoing evolutionary adaptation in host–pathogen interactions. This complexity requires locally tailored solutions that utilize flexible platforms. Future vaccine strategies are unlikely to follow a single universal design; instead, they may require modular approaches that can incorporate multiple antigens. Emerging approaches (such as reverse vaccinology 2.0, machine learning, and mRNA-based platforms) have demonstrated potential for antigen discovery and design. However, their application to ETEC remains largely experimental and requires further validation [82,84,107]. These concepts have been proposed as components of integrative frameworks that combine evolutionary biology with public health considerations, although their practical application for controlling ETEC is still under development.

Future studies should focus on tailoring innovative platforms to specific contexts, such as in Mexico, where diverse ETEC variants, healthcare system inequalities, and advances in biotechnological development coexist [22,61]. Finally, we propose the following research priorities. (1) Characterization of the genomes of circulating ETEC strains in specific regions, with an emphasis on CFAs and resistance mechanisms. In this context, conserved epitope libraries may be computationally explored, including the use of artificial intelligence to optimize candidate multiepitopes. (2) Evaluation of potential and viable vaccines using domestic animal models (piglets or calves) with a One Health approach. (3) Studies on community implementation that combine vaccination with environmental monitoring, education, and sanitation [105]. Clinical trials are a cornerstone of ETEC vaccine development and evaluation. The controlled human infection model (CHIM) has advanced the field by enabling controlled assessments of vaccine efficacy, immunogenicity, and correlates of protection. CHIM studies provide valuable preliminary data; however, their findings require large-scale field trials in endemic settings to ensure external validity. The integration of clinical trials and the CHIM within the ETEC research pipeline is essential for translating findings into effective public health interventions [108].

## Figures and Tables

**Table 1:** Global epidemiological burden of ETEC and its associated mortality and risk factors.

Characteristic	Key Data	Description	Ref(s).
Global prevalence	13–14% of all cases of diarrhea in children aged <5 years	The highest risk was observed in up to 38.2% of children aged 13 to 59 months in a Kenyan cohort.	[1,22,23]
Global mortality	~51,000 deaths in 2016 (~3.7% of diarrhea-related deaths)	Diarrhea-related mortality accounts for 4.7% of all deaths among children under 5 years of age, with variations observed by region.	[1,24]
Most affected age groups	The highest burden is in children aged <5 (peak 6–18 months)	An increased incidence of diarrheal diseases has also been reported in adults aged 20 to 60 years and those over 45 years in endemic areas.	[23,25,26]
Incidence (<5 years)	up to 73 episodes per 100 child-years	Recurrent infections can lead to temporary growth impairment in children.	[8]
Mortality in adults	Increased incidence of severe disease in adults aged >45 years	A significant increase in the number of severe cases in Bangladesh and other endemic regions has been reported.	[25,27]
Risk factor	Poor sanitation increases risk ~3-fold	WASH interventions are critical for reducing the burden of intestinal diseases.	[23,24,26]

This table provides a brief overview of the main global epidemiological indicators related to ETEC infection, including prevalence, mortality rates, age distribution, incidence, and major risk factors. These estimates were derived from multicenter studies and model analyses and may vary on the basis of geographic region, study design, surveillance method, and population characteristics. WASH refers to interventions in water, sanitation, and hygiene.

**Table 2:** Epidemiological and immunological characteristics of ETEC CFAs.

CF	FUP	Morphology		Size (kD)	Host	Serogroup(s)	Toxin(s)
Chaperone-Usher Assembled CFs							
CFA/I-Like Group							
CFA/I	α	F	7 nm	25.0	Humans	O71, O78, O126, O128, O153, ON3	LT, LT + STh, STh
CS1	α	F	7 nm	15.2	Humans	O6, O8	LT + STh
CS2	α	F	7 nm	15.4	Humans	O6	LT + STh
CS4	α	F	6 nm	15.0	Humans	O25	LT + STp
CS14 (PCFO166)	α	F	7 nm	15.0/15.5	Humans	O63, O78, O98, O128, O166	LT + STh, STh
CS17	α	F	7 nm	15.5	Humans	O8, O114, O167	LT
CS19	α	F	7 nm	15.0	Humans	O8, O15, O56, O114	LT + STp
PCFO71	α	n.d.	n.d.	n.d.	Humans		
CS5-like group							
CS5	α	H	5 nm	18.6	Humans	O39, O115, O128, O167	LT + STh, STh
CS7	α	H	3–6 nm	18.7	Humans	O114, O128	LT + STh
Class Ib group							
CS12 (PCFO159)	γ_2_	F	7 nm	17.9	Humans	O159, ON2	LT + STp
CS18 (PCFO20)	γ_2_	F	7 nm	18.5	Humans	O20	
CS20	γ_2_	F	7 nm	17.5	Humans	O9	
CS26	γ_2_	n.d.	n.d.	n.d.	Humans	O64	
CS27A	γ_2_	n.d.	n.d.	n.d.	Humans	O15, O56, OSB16, O160	LT, LT + STp
CS27B	γ_2_	n.d.	n.d.	n.d.	Humans	O56, O179, OSB16, ON13	LT, LT + STp
CS28A	γ_2_	n.d.	n.d.	n.d.	Humans	O159	LT, LT + STp
CS28B	γ_2_	n.d.	n.d.	n.d.	Humans	O15	LT, LT + STp
CS30	γ_2_	F	7 nm	18.5		O9, O9-like, O64	LT + STp
F6 (987P)	γ_2_	F	7 nm	n.d.	Neonatal piglets		LT + STb
Diverse							
CS3	γ_3_	f	2–3 nm	15.0	Humans	O6, O8	LT + STh
CS6	γ_3_	nF		15.1/15.9	Humans	O4, O19, O25, O27, O39, O64O115, O128, O148, O159, O167, O169, O174, O182, ON17	LT, LT + STp, STp
CS15 (Ag 8786)	γ_3_	nF	n.d.	18.2	Humans		n.d.
CS22	γ_3_	f	n.d.	15.0	Humans		n.d.
CS13 (PCFO9)	κ	f	n.d.	24.8	Humans	O9, O64, O112ab, O114, OSB16	LT, LT + STp
CS23	κ	f/nF	n.d.	16.9	Humans	O7, O174-like	n.d.
F4ab/ac/ad (K88)	κ	F	2–4 nm	30.1 (FaeG)	Neonatal and weaned piglets	O6, O149	LT + STb, LT + STp + STb
F7 (F41)	κ	f	3.2 nm	n.d.	Calves, lambs, goat kids, and piglets		n.d.
F5 (K99)	κ	f	3 nm	19.1 (FanC)	Calves, lambs, and goat kids		LT + STb, STp
F18 (F107)	κ	F	6.7 nm	n.d.	Weaned piglets	O8, O147	LT + STb, STp + STb
F17a	γ_4_	f	3–4 nm	n.d.	Calves		LTp, LTIp + STb, STp + STb
CS10 (ag 2230)	n.d.	nF	7 nm		Humans		n.d.
CS11 (PCFO148)	n.d.	f	3 nm		Humans		n.d.
Type IV pili							
CS8 (CFA/III)	-	F	7 nm	25.3	Humans	O8, O25, O27, O169	LT
CS21 (Longus)	-	F	7 nm	25.2	Humans	O6, O8, O25, O27, O71, O78, O126, O128, O148, O169, ON3, ON17,	LT, LT + STh, STh

Table 2 CFAs identified in ETEC strains that infect both humans and animals. This table includes information on their fimbrial usher pathway (FUP) classification, structural morphology, hosts, toxin profiles, and relevance for vaccine development. In humans, the most common strains worldwide are ETEC CFA/I and CS1/CS6, whereas F4 (K88) and F18 are predominant among cases of porcine postweaning diarrhea. Certain CFAs, such as CFA/I-STh and CS6-STp, exhibit consistent associations with specific enterotoxigenic profiles. The diversity of CFAs reflects both evolutionary adaptation and host specificity, highlighting the importance of multivalent vaccine strategies within a One Health framework. F, fimbrial; f, fibrillar; nonfimbrial; H, helical; and n.d., not determined. The sequences and functions of CS10 and CS11 are currently unknown. Only ETEC lineages in which most isolates share the same CFA profile are included. LT refers to either LTlh (human-specific) or LTlp (animal-specific), as adapted from the literature [45].

**Table 3:** Candidates for the development of clinical ETEC vaccines.

Candidate/Name	Technology	Main Targets (Antigens)	Vaccine Platform/Route	Development Phase in Humans (Approx.)	Developer/Company	Reference(s)
ETVAX	Inactivated whole cells + protein subunit	Colonization factors CFA/I, CS3, CS5, and CS6; LT/CT B hybrid (LCTBA)	Oral, inactivated vaccine; optional dmLT mucosal adjuvant	Phase 2 completed in children; Phase 3 planned in LMIC infants and travelers	University of Gothenburg, Scandinavian Biopharma, PATH and partners	[72,73,74,75]
ACE527	Live attenuated whole cells (mixture of 3 strains)	Multiple colonization factors; LT B expression depending on strain	Oral, live attenuated vaccine	Phase 1/2 completed in adults; no recent late-phase trials	Originally Acambis/Sanofi with academic partners (e.g., University of Gothenburg)	[71,73]
MecVax	Multivalent protein subunit (MEFA toxoid + adhesin MEFA)	STa toxoid (3xSTa), LT toxoid (mnLT), CFA/I, and CS1–CS6 adhesins	Injectable intramuscular protein vaccine with adjuvant	Preclinical (protected against diarrhea in mouse and pig models)	Univ. of Illinois Urbana-Champaign, Kansas State Univ., Johns Hopkins	[13,21]
ShecVax (Shigella/ETEC combo)	Multiepitope fusion antigen (MEFA) protein subunit	Shigella antigens (IpaB, IpaD, VirG, etc.) + ETEC STa, LT, CFA/I, and CS1–CS6	Injectable intramuscular protein subunit	Preclinical (showed broad cross-protection in mice, rabbits, and piglets)	Kansas State Univ., University of Illinois Urbana-Champaign, Johns Hopkins	[76]
Adhesin-based recombinant vaccines	Protein subunits (purified adhesins)	Fimbrial “tip adhesins” and major CFs (CFA/I, CS1–CS6, and others)	Mainly parenteral; some exploration of oral/mucosal routes	Mostly preclinical; some candidates in early Phase 1 trials	Multiple academic groups (e.g., Svennerholm and Fleckenstein laboratories)	[7,72,73,77]
LT/ST toxoid-based vaccines	Protein subunits or conjugates (LT and ST toxoids)	LT toxoid, ST toxoid alone or fused to carrier proteins/nanoparticles	Injectable subunit, nanoparticle; candidate mRNA and glycoconjugates in early work	Preclinical (toxin neutralization in animal models)	International academic consortia (including Norway/Bergen and others)	[7,13,21,78]
MEFA-based adhesin platforms (new designs)	Multiepitope fusion antigen (MEFA)	Multiple conserved adhesin epitopes; sometimes includes LTb as a built-in adjuvant	Protein subunit, intramuscular; conceptual mRNA formats	Preclinical (mice were protected against a lethal challenge)	Universities in China and other countries	[13,21,79]
Vaccines with novel conserved antigens	Recombinant protein subunits	EtpA, EatA, YghJ, and other conserved autotransporters, plus flagellin	Mainly parenteral; often combined with CFs and/or toxoids	Preclinical; strong immunogenicity and the production of functional antibodies in animals	Fleckenstein, Qadri and collaborators	[7,21,77]

This table summarizes the status of ETEC vaccine candidates in clinical development. MEFA (multiepitope fusion antigen), CFs (colonization factors), heat-labile toxin (LT), heat-stable toxin (ST), mRNA, colonization factor antigen I (CFA/I), Coli surface antigen (CS), invasion antigen B (IpaB), and enteroinvasive *E. coli* (EIEC).

## Data Availability

The original contributions presented in this study are included in the article/Supplementary Material. Further inquiries can be directed to the corresponding authors.

## Supplementary Materials

The following supporting information can be downloaded at: https://www.mdpi.com/article/10.3390/microorganisms14061171/s1, Figure S1. Flowchart depicting the literature search and study selection process for research on ETEC, CFA, vaccines, genomics, and One Health.
