# Supplementary figures and images for: One Health Perspective on the Enterotoxigenic Escherichia coli Diversity
Source: Microorganisms. 2026 May 22;14(6):1171. doi: 10.3390/microorganisms14061171 (PMC13303640; doi:10.3390/microorganisms14061171)

# Search and Study Selection: ETEC, CFAs, Vaccines and One Health

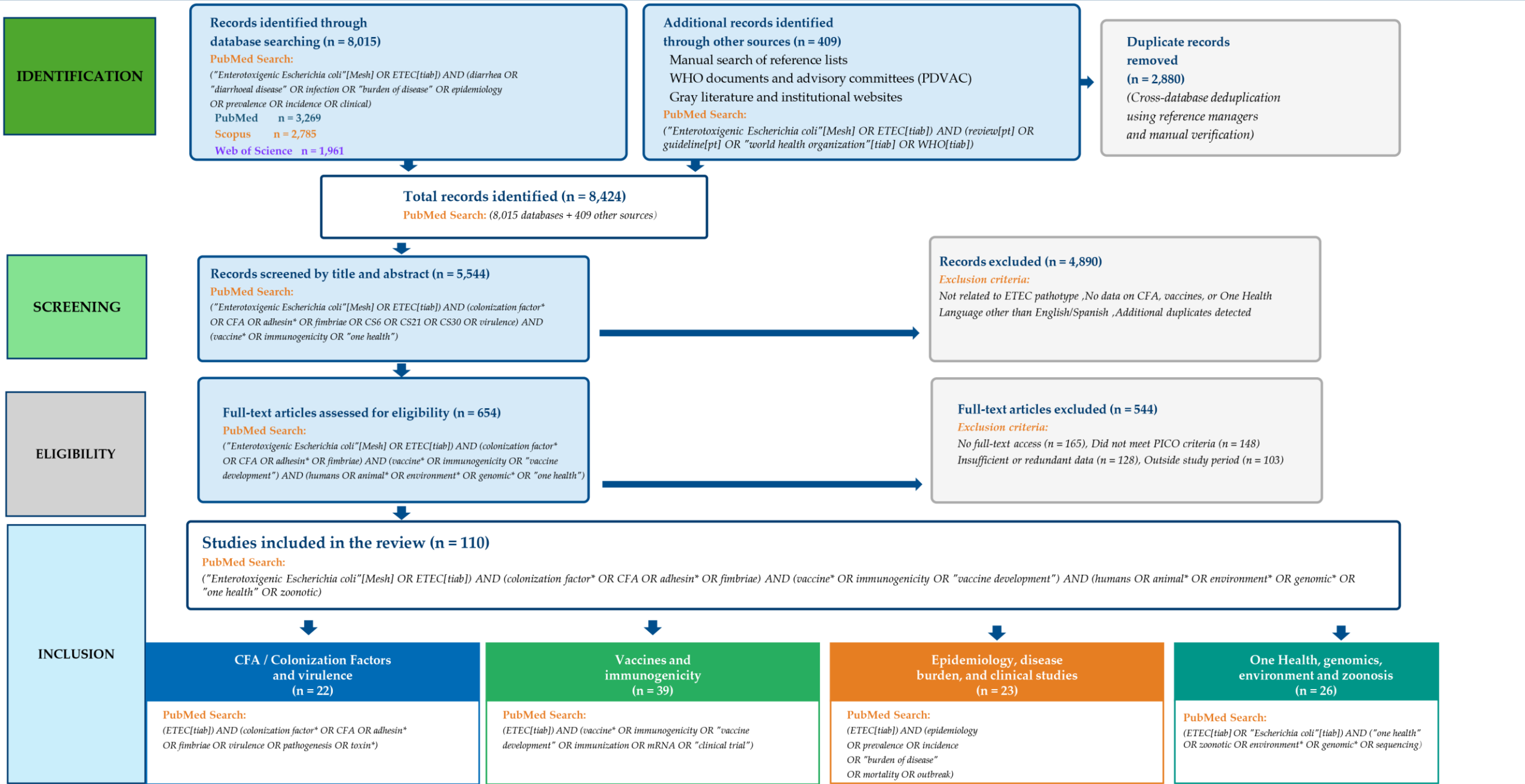

Supplement: Supplementary file 1 [file microorganisms-14-01171-s001.zip › microorganisms-4292441-supplementary.pdf]
